# Influence of forehead gap size on eye protection provided by lead eyewear worn over prescription glasses: A Monte Carlo study

**DOI:** 10.1002/mp.70734

**Published:** 2026-10-25

**Authors:** Ryota Shindo, Masaki Fujisawa, Keisuke Yamamoto, Kodai Sagehashi, Yohei Inaba, Koichi Chida

**Affiliations:** ^1^ Department of Radiological Technology Tohoku University Graduate School of Medicine Sendai Japan; ^2^ Division of Radiological Disasters and Medical Science Department of Disaster Medicine International Research Institute of Disaster Science Tohoku University Sendai Japan

**Keywords:** interventional radiology (IR), Monte Carlo simulation, Pb glasses (lead eyewear), radiation protection

## Abstract

**Background:**

Due to exposure to high levels of scattered ionizing radiation, interventional radiology (IR) physicians have an elevated risk of radiation‐induced eye injuries and cataracts. Previous studies have evaluated the protective effects of regular lead glasses and recommended measures to improve radiation protection for the eyes. However, the protective effects of fit‐over eyewear—lead eyewear worn over prescription glasses—have not been thoroughly examined. Importantly, a previous study noted that the space required to accommodate prescription glasses may reduce eye protection.

**Purpose:**

Monte Carlo (MC) simulations were performed to quantify the protective effects of fit‐over eyewear and assess the relative effectiveness of adding bottom shields to conventional lead eyewear to improve eye protection.

**Methods:**

Conventional lead glasses comprising front and side lenses and prescription glasses with plastic lenses and titanium frames were modeled. Lead glasses with bottom shields were created by bending the lower edges of the front lenses of conventional eyewear without increasing the total lens area. Three lead lens thickness values were tested: 0.07, 0.5, and 0.75 mm. Using a mesh‐type reference computational phantom representing an adult male to simulate an IR physician, MC simulations were performed with a transcatheter arterial chemoembolization setup. The Particle and Heavy Ion Transport code system was used to calculate the radiation dose deposited in each eye‐sensitive volume. The distance (*d*) between the forehead and front lead lens varied systematically. Protective effects were evaluated using the shielding rate (%) calculated as: (1‐*D_pr_*/*D*_0_) × 100, where *D*_0_ is the radiation dose without protection and *D_pr_* is the dose with eye protection.

**Results:**

For the eye near the X‐ray source (left eye), the conventional models showed shielding rates >50% when the lead thickness was 0.5 or 0.75 mm and *d* was ≤0.5 cm. The conventional 0.07‐mm lead eyewear exhibited shielding rates <50% when *d* was 0.5 cm, and <26% when *d* was ≥0.8 cm. However, 0.07‐mm lead eyewear with bottom shields maintained approximately 50% shielding when *d* increased to 1.0 cm. The models using 0.5‐ or 0.75‐mm lead lenses with bottom shields exhibited shielding rates >76% at *d* = 1.0 cm. Notably, the shielding rates for the right eye were <12% regardless of introducing bottom shields. The right eye doses were 45–48% of the left eye lens dose without protection.

**Conclusion:**

By quantifying the relationship between the size of the lens gap and shielding rates, this study estimates the protective effects of fit‐over eyewear and can help IR physicians select suitable eyewear. Our findings show that fit‐over eyewear with bottom shields effectively protects the left eye during posterior‐anterior projection. Although introducing bottom shields can improve conventional side‐shield models in terms of increased shielding rate for the left eye, the shielding rates for the right eye remained low because of the gap around the nose. Further study of new designs with a shielded covering over the nose may be warranted.

## 1 INTRODUCTION

Interventional radiology (IR) procedures are widely used because of their low invasiveness and broad applicability.^1^, ^2^ However, prolonged and repeated fluoroscopic procedures can cause radiation‐induced skin injuries in patients^3^, ^4^, ^5^ and result in IR physicians being exposed to high levels of scattered radiation.^6^, ^7^, ^8^, ^9^, ^10^, ^11^, ^12^, ^13^, ^14^ As a result, IR physicians face elevated risks of radiation‐induced eye injuries and cataracts.^15^, ^16^, ^17^, ^18^, ^19^, ^20^, ^21^

In April 2011, the International Commission on Radiological Protection (ICRP) lowered the threshold dose for cataracts to 0.5 Gy and recommended reducing the new occupational equivalent dose limit for the eye lens to “20 mSv in a year, averaged over defined periods of 5 years, with no single year exceeding 50 mSv.^22^, ^23^” Given these new guidelines, there is increasing need to improve radiation protection for the eyes available for IR physicians.^24^, ^25^, ^26^, ^27^

During IR procedures, the ceiling‐suspended screen shields the primary operator from most scattered photons.^28^, ^29^, ^30^ However, the protective effect may be reduced if the shield is not properly positioned. For this reason, it is recommended that the screen is combined with lead (Pb) eyewear to ensure adequate eye protection. Several studies have evaluated the level of eye protection afforded by regular Pb eyewear in clinical settings, phantom experiments, and Monte Carlo (MC) simulations.^9^, ^31^, ^32^, ^33^, ^34^, ^35^, ^36^ However, few studies have examined the protective effects of Pb eyewear worn over prescription glasses.

Previous phantom studies have evaluated protection provided by such fit‐over eyewear with 0.75 mm Pb front lenses and 0.5 mm Pb side shields.^37^, ^38^ However, these studies noted several limitations, including gaps between the Pb lenses and the head, and the heavy weight of Pb lenses thicker than 0.5 mm. The gaps are a particular concern, as scattered photons incident from the side and below may pass through these gaps and directly reach IR physicians’ eyes. Notably, prescription glasses were not worn in these phantom studies, and so the protective effects of the prescription glasses themselves were not accounted for.

In response to these findings, phantom studies were conducted using an alternative option: fit‐over eyewear equipped with 0.07 mm Pb‐equivalent bottom shields.^39^, ^40^ These studies irradiated a phantom representing an operator's head with primary X‐rays and found that the fit‐over eyewear shielded approximately 70% of X‐rays incident from the left side (i.e., the X‐ray source side) and below. These findings support the effectiveness of bottom shields and large lenses in improving protective effects. Eye protection was also improved when fit‐over eyewear was worn over plastic prescription glasses, suggesting photon attenuation by the front frame of the prescription glasses. However, the effectiveness of 0.07 mm Pb bottom shields in clinical settings remains unclear. Moreover, while it is known that protection is decreased when Pb eyewear does not fit well,^41^ the relationship between the gap size and protective effect of fit‐over eyewear has not been investigated. As the distance between the Pb lenses and head may vary depending on the combination of fit‐over eyewear and prescription glasses, it is necessary to consider the reduction in protective effects caused by larger gaps and potential photon attenuation provided by prescription glasses.

To address these limitations of previous studies, we conducted MC simulations modeling a clinical setup. In these simulations, the fit‐over eyewear was worn over prescription glasses, and the distance between the Pb lens and forehead was systematically varied. We also created fit‐over glasses with bottom shields of different Pb thicknesses and compared their protective effects with those of conventional side‐shield models. This approach allowed us to evaluate the protective effects of widely used eyewear models while considering practical fit conditions and clarifying any enhanced protective effects of bottom shields.

## 2 METHODS

This study used MC simulations to quantify the protective effects of various forms of protective eyewear under varying conditions. The simulations used standard values for the weight fractions and densities of water and simple substances. For other compounds, we used the values listed in data tables provided by the Pacific Northwest National Laboratory,^42^ unless otherwise specified.

### 2.1 Pb eyewear models

Two types of Pb lenses were modeled: (i) a conventional Pb lens model consisting of front and side lenses (FSL model; Figure 1a) and (ii) a front‐side‐bottom lens model (FSBL model, Figure 1b). The material of these Pb lenses was defined as pure Pb. The sizes and shapes of Pb lenses were generally in accordance with Panorama Shield HF‐480S (Toray, Tokyo, Japan),^40^ with partial modifications for simplification and integration into the human phantom head. The FSBL model was created by bending the front lens of the FSL model at a 45°angle. The total lens area of FSBL model was approximately 164 cm^2^, which is 0.49% smaller than that of the FSL model. The frames and temples of the Pb glasses were not modeled. For each design, Pb lenses with thicknesses of 0.07, 0.5, and 0.75 mm were created. The Pb eyewear models were used in combination with prescription glasses, except for the simulations evaluating the shielding effects of prescription glasses (see Section 2.5).

**FIGURE 1:**
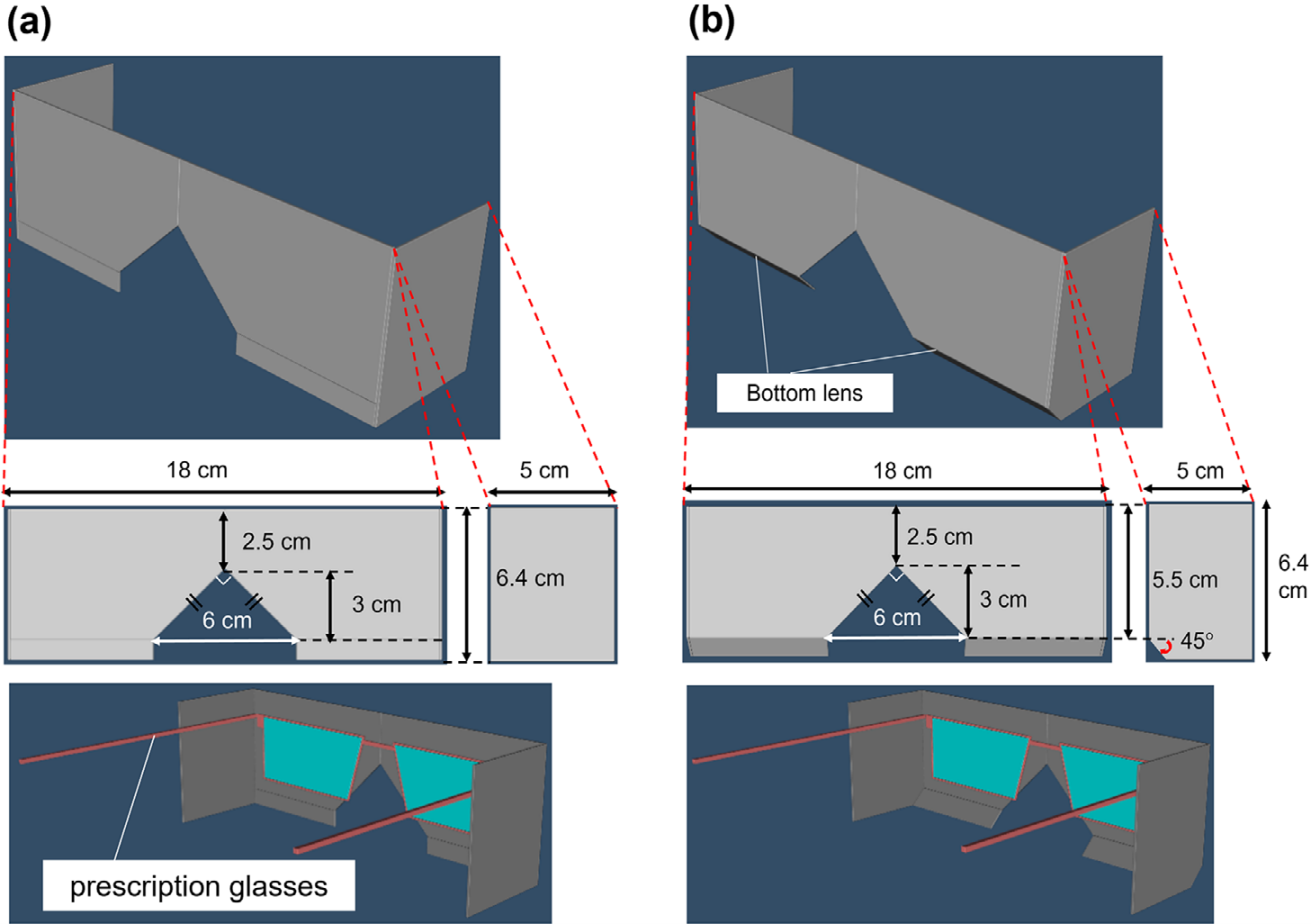
Two different shapes of Pb lenses were combined with prescription glasses (a: FSL model; b: FSBL model).

### 2.2 Prescription glasses model

Figure 2 presents the prescription glasses used in this study. The design was based on commercial prescription glasses (model number MF67‐054; brand: Maxim flex; manufacturer: HASEGAWA Co., Ltd., Fukui, Japan) with frame and lenses made of titanium and acrylic resin, respectively. Titanium is a commonly used metal for eyewear frames because of its light weight, high strength, and good corrosion resistance.^43^ Lenses are typically made of plastic materials, which are lighter, thinner, and more impact‐resistant than glass.^44^, ^45^ The lenses used in this study had a thickness of 0.1 cm, which is classified as a thin lens.^46^

**FIGURE 2:**
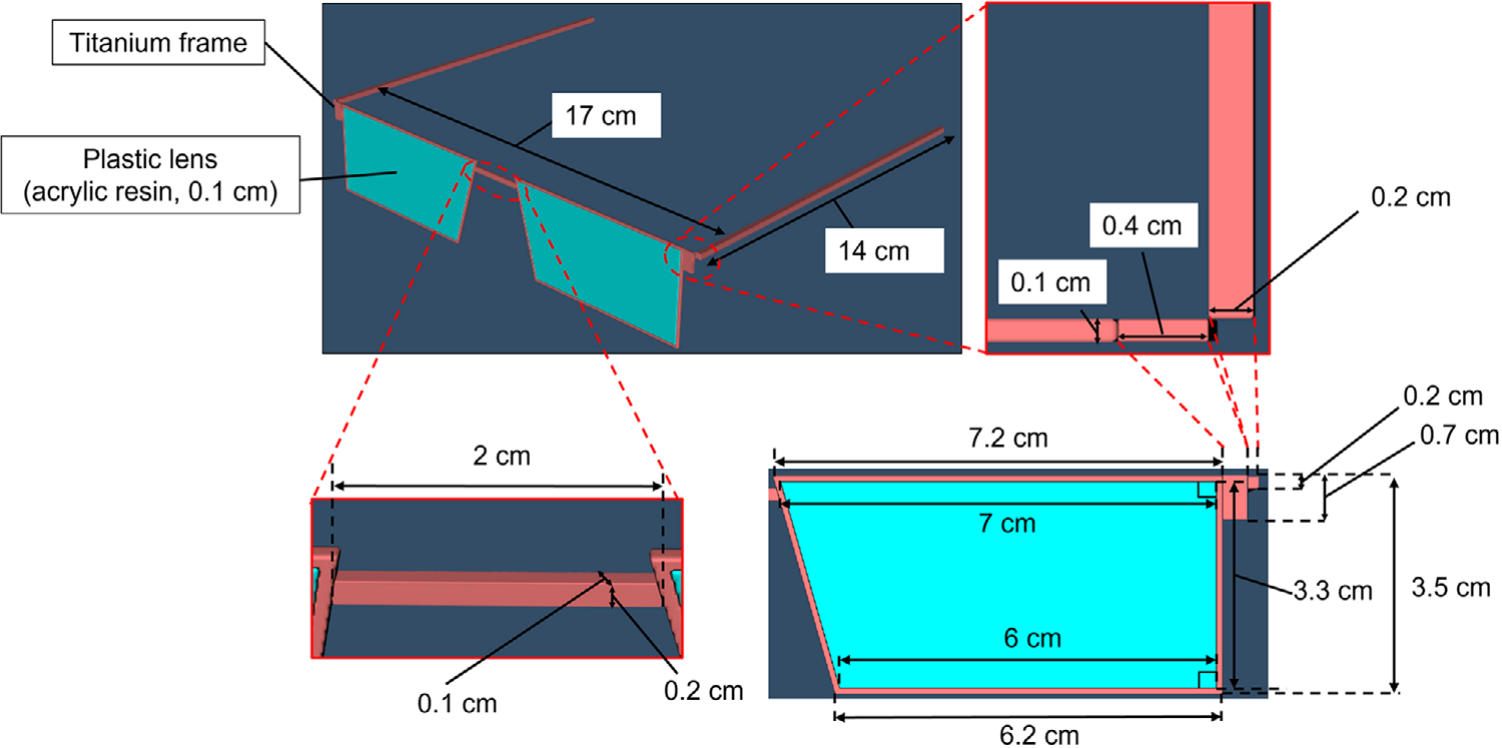
Design of the prescription glasses. The lenses and frame were made of plastic and titanium, respectively.

### 2.3 Human phantom

Our simulations used the mesh‐type reference computational phantom representing an adult male (MRCP‐AM)^47^ constructed by converting the voxel phantoms reported in ICRP Publication 110 into a high‐quality mesh format. The MRCP‐AM was wrapped in continuous skin and incorporated detailed eye models described by Behrens et al.^48^, ^49^ This MRCP‐AM represents the contours and surface features of the human head more precisely than other models and is thus considered one of the most appropriate models for MC simulations of the fit of Pb eyewear to the phantom's head.

### 2.4 Eye lens dose calculations

Several previous studies have used the kerma approximation to calculate the dose deposited in the radiation‐sensitive volume of the eye lens.^50^, ^51^, ^52^ The kerma approximation assumes that the energy of secondary electrons is deposited locally.^53^ This simplification enables faster computation. Therefore, we first confirmed the validity of the kerma approximation for eye lens dose calculations using the MRCP‐AM phantom. To do so, we compared doses calculated using the kerma approximation with those calculated by transporting secondary electrons.

The MC transport calculations were conducted using the Particle and Heavy Ion Transport code system (PHITS) version 3.350.^54^, ^55^ PHITS is a general‐purpose MC code that can handle a variety of particles over a wide energy range. PHITS calculates absorbed dose as an expected value per primary particle (e.g., Gy/photon). For the kerma approximation, only photons with energies at or above 1 keV were transported based on the original PHITS algorithm. In contrast, the calculations considering secondary electrons, performed using the EGS5 model included in the PHITS code (PHITS‐EGS),^56^ tracked both photons and electrons with energies at or above 1 keV.

According to the validation method described by Behrens et al.,^57^ the MRCP‐AM phantom was placed in a vacuum, and a monoenergetic parallel photon beam covering the entire body region from the feet to the head was incident on the phantom. The anterior‐posterior (AP) and left‐right (LR) directions were chosen as the beam directions because the sources of scatter radiation (e.g., the patient's irradiated body part and X‐ray beam‐limiting devices) were positioned in front of and to the left of the IR physician.^38^ The incident photon energies varied from 10 to 120 keV. The deposition energies in the left eye‐sensitive volume were then calculated. In the PHITS output file, statistical error is expressed as the ratio of the standard error to the mean. MC simulations for the validations continued until this ratio achieved 5% or lower. MC simulations were performed on a desktop computer equipped with an Intel Core Ultra 7 265KF processor (3.90 GHz, 20 cores) and 128 GB RAM running Windows 11 Pro (64‐bit).

Figure 3 presents the comparison of the two different dose calculation methods. For AP irradiation, the doses calculated using the kerma approximation tended to be slightly lower than those calculated using PHITS‐EGS, particularly at energies below 30 keV. A similar trend toward underestimation has also been reported for skin dose calculations in the diagnostic energy range.^53^ However, in the eye lens dose calculations in this study, the differences between the kerma approximation and PHITS‐EGS were small, ranging from −9.8% to 4.2% for AP irradiation and from −0.86% to 13% for LR irradiation. Therefore, no substantial underestimation was observed in the diagnostic energy range (≤120 keV). In addition, the number of histories required for PHITS‐EGS was on the order of 10^8^–10^9^, which was 10–100 times that for the kerma approximation. Under an execution condition using 18 CPU cores, the computation time for the 80 keV AP irradiation geometry was approximately 2.5 min for the calculation based on the kerma approximation, whereas the calculation using PHITS‐EGS5 required approximately 9.4 h. Given these findings, we determined that the kerma approximation was suitable for dose calculations of eye lens‐sensitive volumes.

**FIGURE 3:**
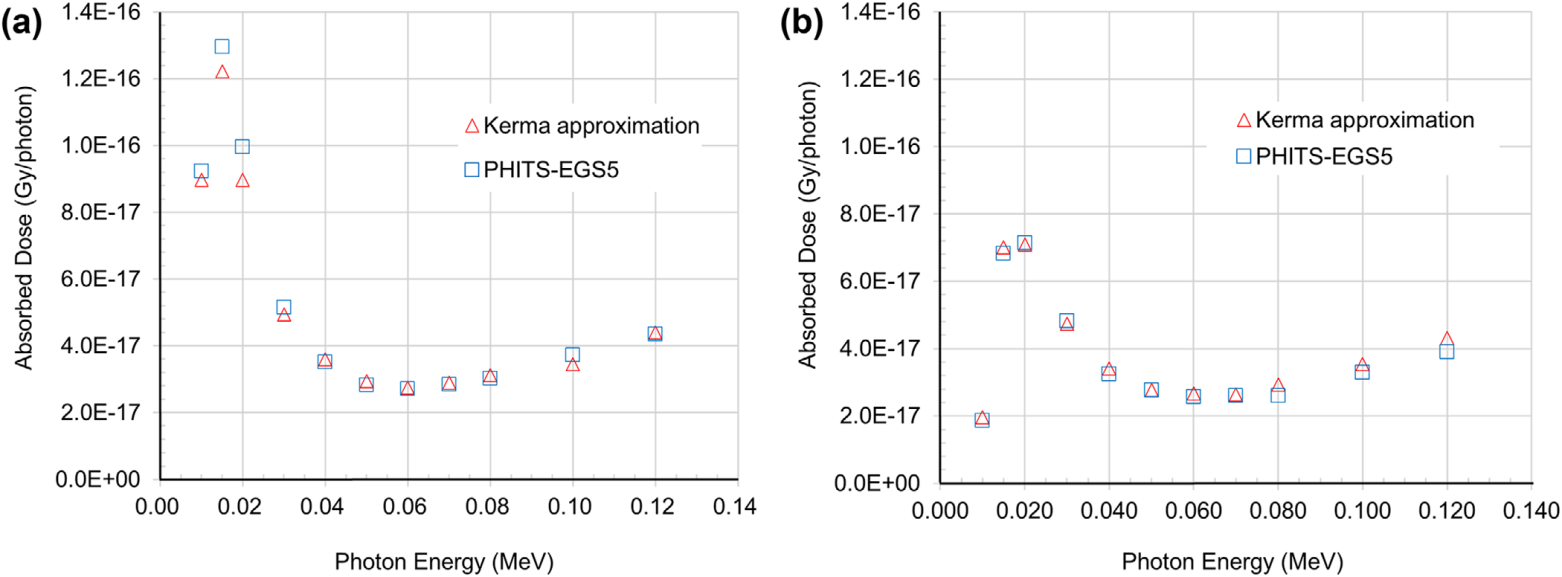
Comparison of the left eye lens dose using two different calculation methods (a: AP irradiation; b: LR irradiation). Absorbed dose was calculated as an expected value per primary photon. X‐axis represents monoenergetic photon energy. Statistical errors in dose calculations were maintained at ≤5%.

### 2.5 MC simulations using IR theater geometry

Figure 4 shows the IR theater geometry used in our MC study, which was modeled on the transcatheter arterial chemoembolization (TACE) setup described by Mao et al.^52^ The IR operator was represented by the MRCP‐AM. The patient's abdomen was simply modeled as a water cylinder, with dimensions corresponding to those of an abdominal CTDI phantom, without any holes for placing detectors. The patient couch was modeled as carbon with a density of 1.93 g/cm^3^, based on that of carbon fiber.^58^, ^59^ The IR theater was modeled as filled with air.

**FIGURE 4:**
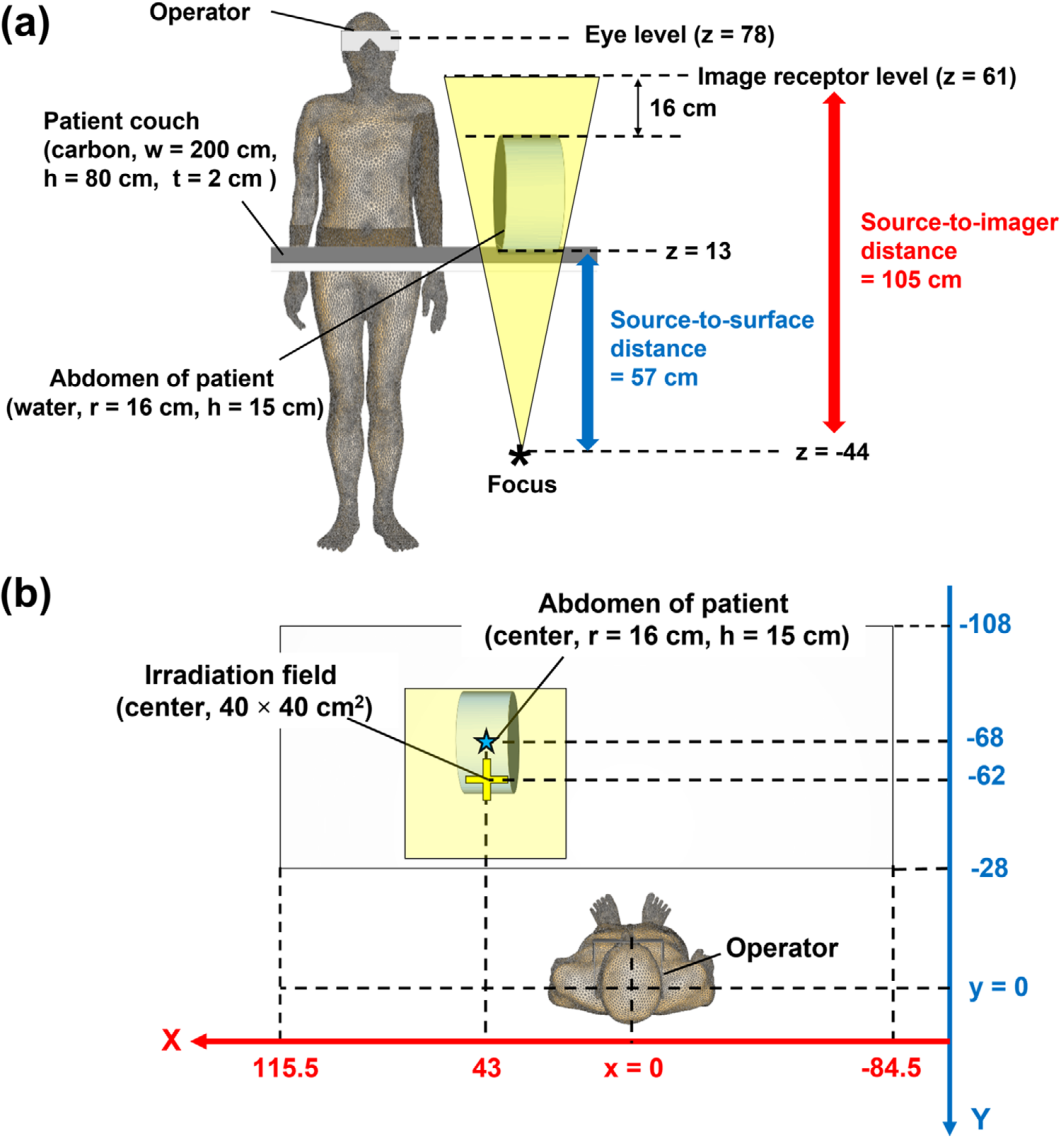
Diagram of the simulation geometry (a: front view; b: top view).

The X‐ray tube was simplified as a point source and collimated by an outer‐void frame, in which all calculations of particles entering it were terminated. The X‐ray source was located beneath the patient couch, with the patient irradiated from the posterior‐anterior (PA) direction. As the purpose of this study was to evaluate eye protection against scattered radiation produced in the patient's irradiated abdomen, the X‐ray beam‐limiting devices and the image receptor were not modeled. The source‐to‐surface distance was set to 57 cm. The source‐to‐imager distance was assumed to be at 105 cm, and the irradiation field size was set to 40 × 40 cm^2^ at this distance.

The X‐ray energy spectrum was calculated using SpekPy version 2.5.3, which is Python software for modeling X‐ray spectra from X‐ray tubes.^60^, ^61^, ^62^ Using this software, the X‐ray tube voltage, additional filtration, and anode angle were set to 80 kVp, 2.9‐mm Al, and 8°, respectively. For the physics model, kqp (most accurate model) was chosen. Figure 5 shows the X‐ray energy spectrum made with SpekPy. Heel effects were not accounted for in our MC simulations.

**FIGURE 5:**
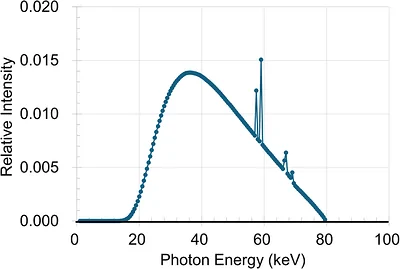
X‐ray spectrum used in this study. The intensities of each energy bin were normalized such that the total integrated intensity over the energy spectrum is unity (as implemented in PHITS calculations).

Figure 6 illustrates the position of the Pb eyewear and prescription glasses in the MC simulations. The prescription glasses were placed 0.3 cm from the most protruding point of the forehead in the sagittal plane at *x* = 0 cm. For the Pb eyewear, the distance (*d*) between the front Pb lens and forehead was set as 0.5, 0.8, 1.0, 1.3, 1.5, 1.8, or 2.0 cm.

**FIGURE 6:**
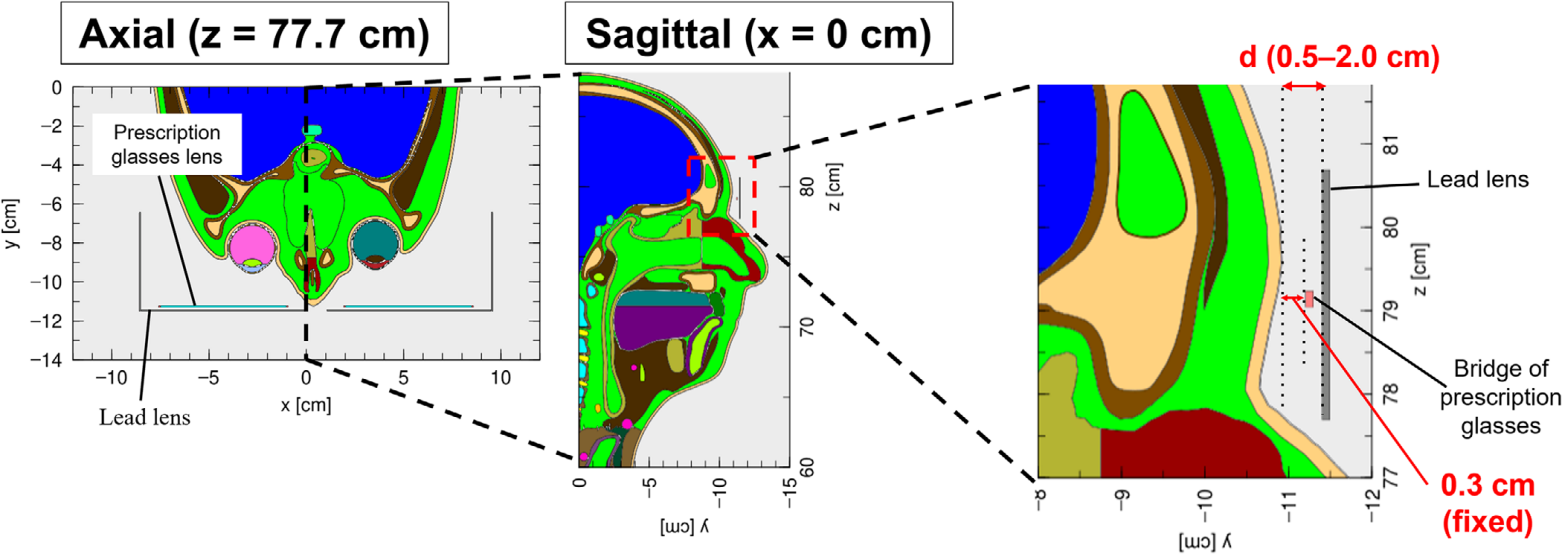
Positioning of the Pb eyewear and prescription glasses. Due to slight asymmetry in the phantom head, the eyewear was shifted 0.5 cm along the x‐axis to avoid contact with the nose.

Eye protective effects were evaluated using the shielding rate (%) calculated as follows:

$$\mathrm{Shielding}\;\mathrm{rate}\;=\left(1-\frac{D_{pr}}{D_{0}}\right)\times 100$$

where *D*_0_ is the dose of the eye lens without protection and *D_pr_* is the dose of the protected eye lens. Dose calculations were conducted until statistical error reached 3.5% or lower. Thus, the statistical errors of the shielding rate propagated from the dose uncertainties were below 5%. Since this study focused on relative comparisons of protective effects, the simulations were conducted using a simplified system as described above, and no experimental measurements were performed. Therefore, the calculated doses were not normalized to clinical dose units (i.e., Gy or Sv).

In addition, the shielding rates were calculated with the phantom wearing Pb eyewear or prescription glasses to evaluate the contribution of prescription glasses. For these simulations, the distance between the front Pb lens and forehead was fixed at 0.5 cm.

### 2.6 Use of generative AI

ChatGPT (OpenAI, GPT‐4.1) was used for grammar and language editing in preparing this manuscript. All content was reviewed and verified by the authors, who take full responsibility for it.

## 3 RESULTS

### 3.1 Impact of the gap between the forehead and front Pb lens on eye protection

Figure 7 shows changes in shielding rates for the left eye with an increasing distance (*d*) between the forehead and the front Pb lens. The results showed little difference in the shielding rates between the 0.5 mm and 0.75 mm lenses. The FSBL model with 0.07 mm Pb thickness provided higher shielding rates than the FSL models with 0.5 and 0.75 mm Pb thickness. Specifically, the FSL model showed shielding rates >50% with a Pb lens thickness of 0.5 or 0.75 mm and *d* = 0.5 cm, while the FSBL model maintained shielding rates of nearly 50% or higher up to *d* = 1.0 cm for 0.07 mm Pb thickness and up to *d* = 1.3 cm for 0.5 and 0.75 mm Pb thickness. The FSBL models with a Pb thickness of 0.5 and 0.75 mm also showed high protective effects of >76% up to *d* = 1.0 cm.

**FIGURE 7:**
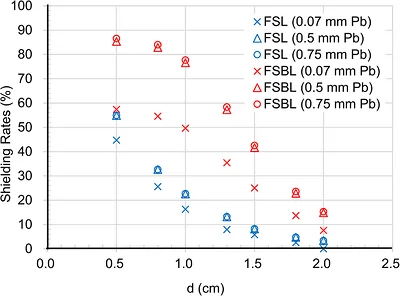
Relationship between left eye protection and the distance (*d*) between the forehead and front Pb lens. All data were calculated with prescription glasses worn.

Figure 8 shows the shielding rates for the right eye. The values were <12%, indicating that the right eye was not efficiently protected. Notably, the shielding rates were very similar across models and remained low regardless of distance between the front Pb lens and the forehead. Although the shielding rates increased with increasing Pb thickness, the magnitude of the increase was limited.

**FIGURE 8:**
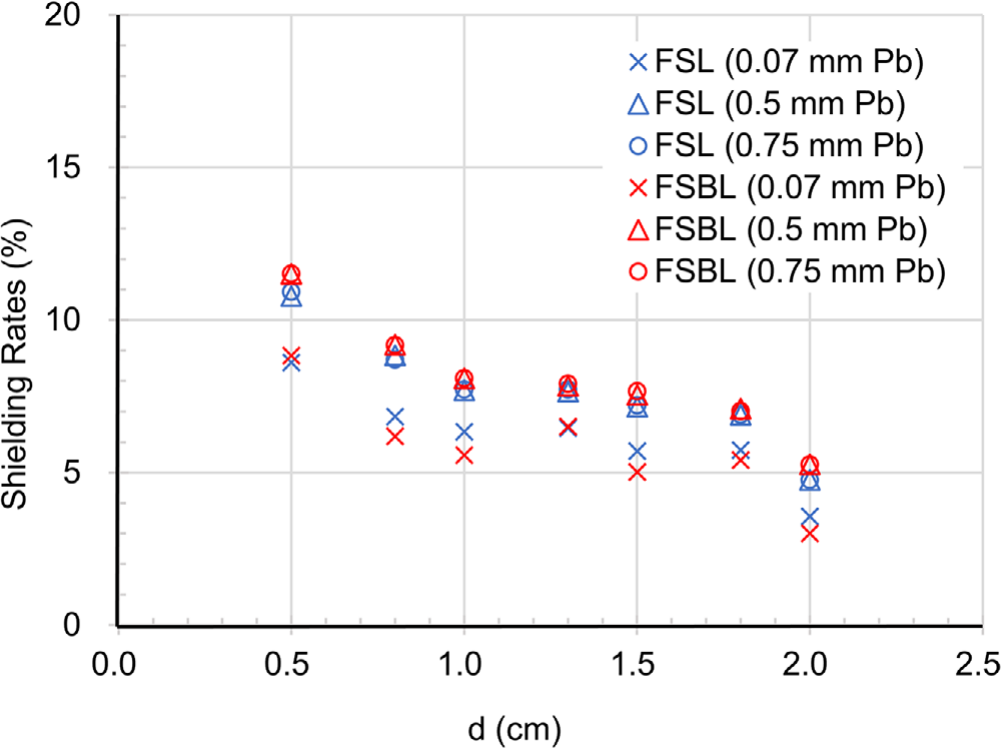
Relationship between right eye protection and the distance (*d*) between the forehead and front Pb lens. All data were calculated with prescription glasses worn.

### 3.2 Comparison of left and right eye lens doses

Figure 9 presents the protected eye lens doses normalized to the left eye lens doses without protection. With a decreasing distance, the left eye lens doses decreased and approached the right eye lens doses. In these simulations, the right eye doses were 45–48% of the left eye lens dose without protection. For the FSL models, the left eye lens doses were higher than right eye when *d* ≥ 0.8, and were comparable to or higher than the right eye dose at *d* = 0.5 cm (Figure 9a). However, for the FSBL model with a Pb thickness of 0.5 or 0.75 mm, the left eye lens doses were lower than the right eye doses when the distance decreased to 1.3 cm or less (Figure 9b).

**FIGURE 9:**
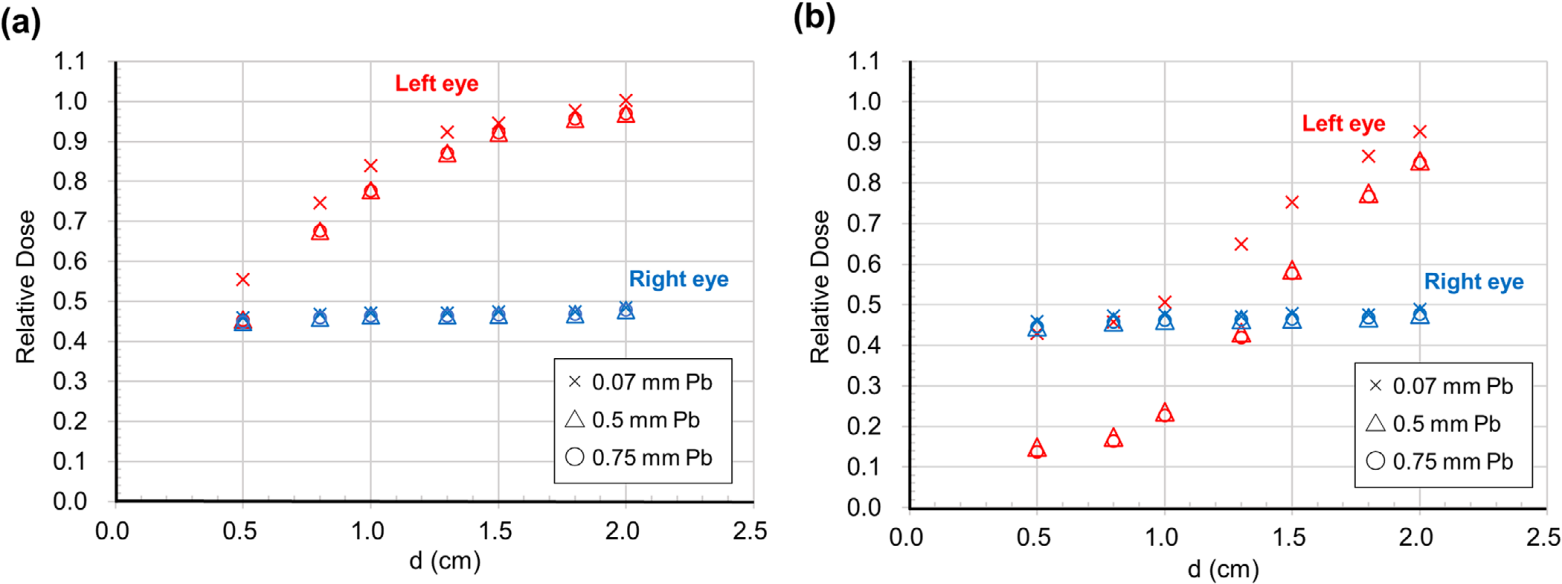
Eye lens dose calculated for Pb eyewear combined with prescription glasses (a: FSL model; b: FSBL model). Data are normalized to the left eye dose without protection.

### 3.3 Spatial distribution of scattered photons

Figures 10 and 11 illustrate the spatial distribution of scattered photon fluence on several x–y and y–z planes for *d* = 1 cm. The scattered photons were mainly distributed on the left side, as the X‐ray source was located on the left side of the operator. The left eye was protected by both the front and side shields, and only a small number of photons reached the prescription glasses (Figure 10). In addition, the FSBL model further reduced the scattered photon fluence incident on the left eye owing to the bottom shield (Figure 11b). In contrast, for the right eye, scattered photons reached the eye through the gap around the nose region regardless of the presence of the bottom shield (Figures 10 and 11). In addition, scattered photons were observed to be incident on the right lens and frame of prescription glasses at *z* = 77 and 78 cm (Figure 10).

**FIGURE 10:**
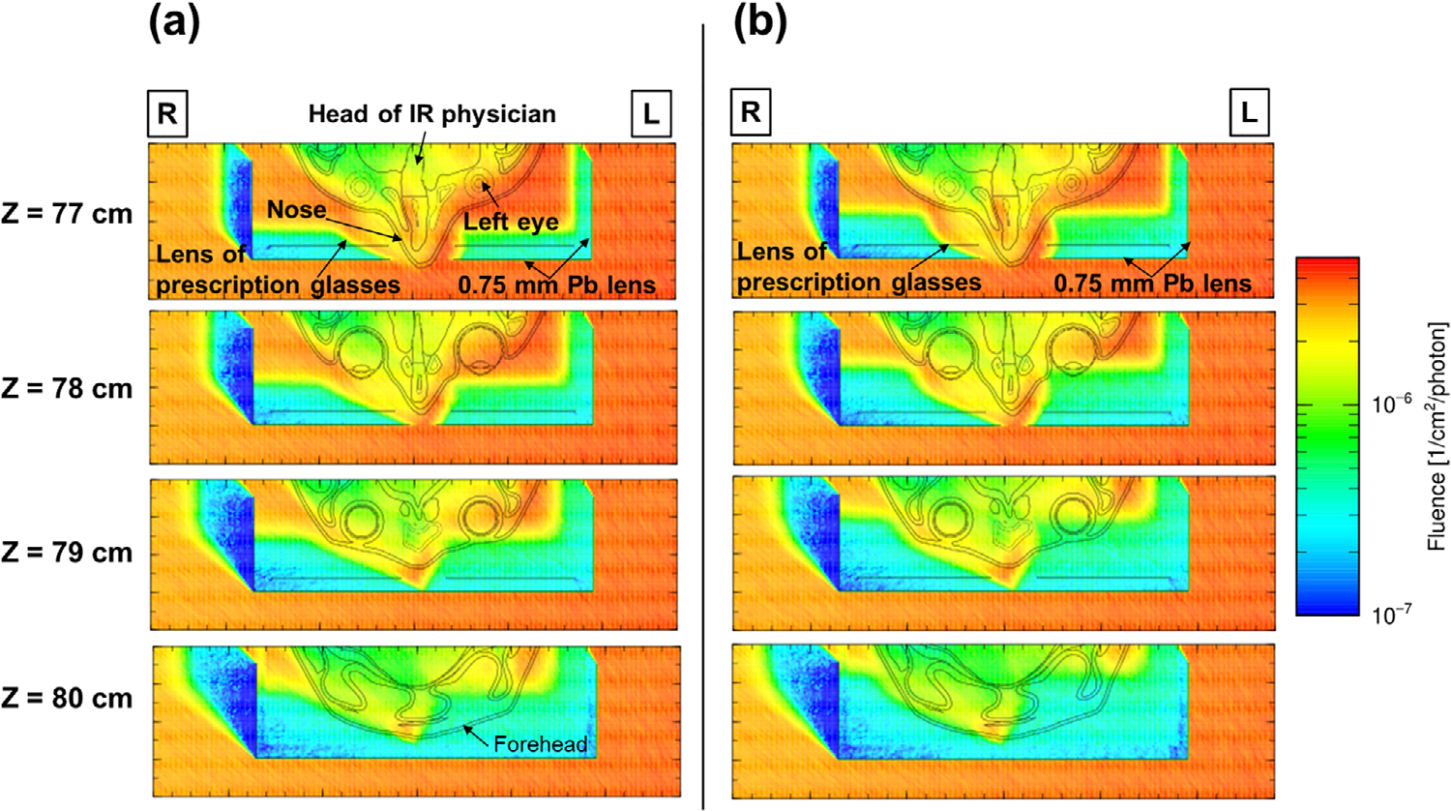
Spatial distributions of scattered photons incident on IR physician wearing 0.75 mm Pb glasses and prescription glasses (a: FSL model; b: FSBL model). In this study, the sources of scattered photons (i.e., the patient body and X‐ray source) were located on the left side of the physician. The distance (*d*) between the forehead and the front Pb lens was set to *d* = 1.0 cm.

**FIGURE 11:**
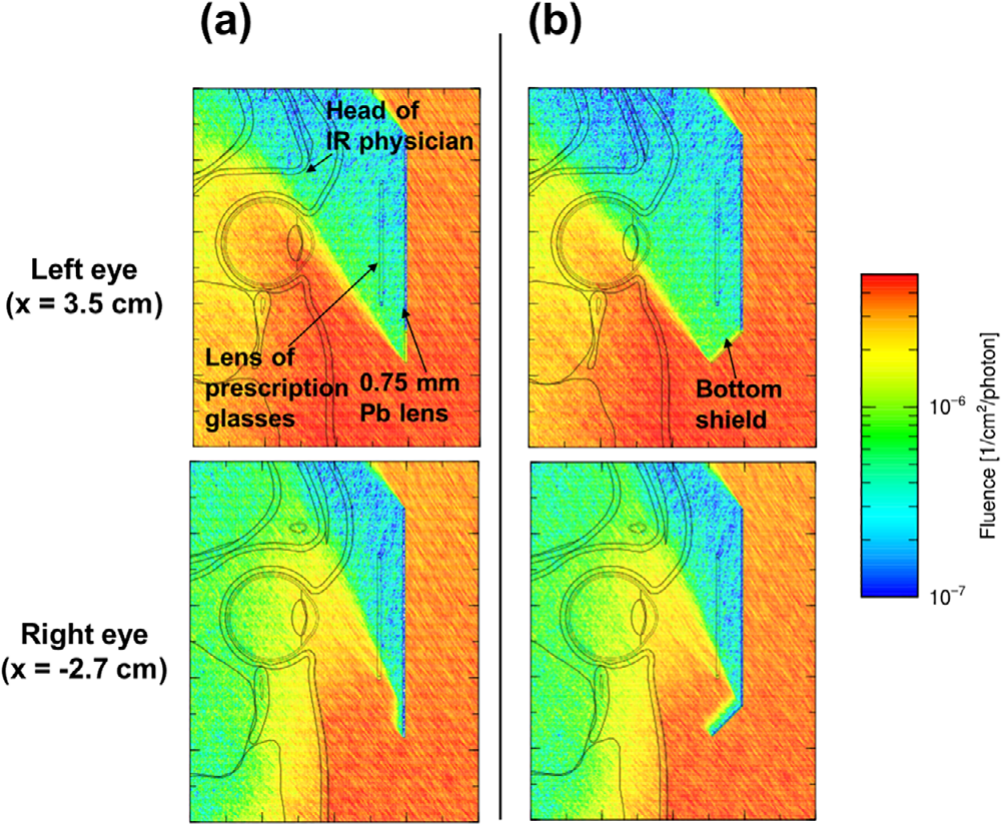
Spatial distributions of scattered photons on the y–z planes at *x* = −2.7 and 3.5 cm, corresponding to the right and left eyes, respectively (a: FSL model; b: FSBL model). The distance *d* was set to 1.0 cm. The thickness of the Pb lenses was 0.75 mm.

### 3.4 Eye lens protective effect of prescription glasses

Tables 1 and 2 show the shielding rates of Pb eyewear with and without prescription glasses, as well as the shielding rates for prescription glasses. The distance between the forehead and the front Pb lens was maintained at 0.5 cm. For the prescription glasses, the distance between the bridge and the forehead was maintained at 0.3 cm. For the left eye, there was little difference in the shielding rates of Pb eyewear with or without prescription glasses. Although right eye protection tended to increase when prescription glasses were worn, the shielding rate of prescription glasses alone was only 3.4%.

**TABLE 1:** Comparison of left eye protection with and without prescription glasses. The distance between the forehead and front Pb lens was maintained at 0.5 cm.

		Shielding rates (%)	
Usage status of lead glasses	Pb lens thickness (mm)	With prescription glasses	Without prescription glasses
FSL model	0.07	44	45
	0.5	55	55
	0.75	55	55
FSBL model	0.07	57	58
	0.5	85	85
	0.75	86	86
Without Pb glasses	N/A	−0.33	N/A

*Note*: The distance between the forehead and front Pb lens was maintained at 0.5 cm.

**TABLE 2:** Comparison of right eye protection with and without prescription glasses. The distance between the forehead and front Pb lens was maintained at 0.5 cm.

		Shielding rates (%)	
Usage status of lead glasses	Pb lens thickness (mm)	With prescription glasses	Without prescription glasses
FSL model	0.07	8.6	5.2
	0.5	11	8.2
	0.75	11	8.3
FSBL model	0.07	8.8	5.3
	0.5	12	8.7
	0.75	12	8.7
Without Pb glasses	N/A	3.4	N/A

*Note*: The distance between the forehead and front Pb lens was maintained at 0.5 cm.

## 4 DISCUSSION

Conventional fit‐over Pb eyewear has a gap at the bottom to allow it to be worn over prescription glasses.^37^, ^38^ If the Pb eyewear does not fit properly, additional gaps may be created. To address eye protection under these conditions, this study used MC simulations to examine the protective effects with an increasing distance between the forehead and front Pb lens. We also examined the effectiveness of bottom shields to enhance eye protection when using fit‐over eyewear. By doing so, this study provides useful information for selecting appropriate fit‐over Pb eyewear. Our findings support that conventional side‐shield models can be improved by introducing bottom shields without increasing the total lens area or Pb equivalent thickness.

A previous study reported that the left eye dose during TACE procedures, using only a ceiling‐suspended shielding screen without protective eyewear, was 240 µSv per procedure. This corresponds to a maximum of 83 procedures per year without exceeding the 20 mSv/year dose limit. Therefore, the use of lead glasses and further improvement of eye protection during TACE procedures are considered important issues.^63^

### 4.1 Impact of the gap between the forehead and front Pb lens

Our evaluation indicated that FSBL models—which were created by bending the front lenses of the FSL model without increasing the total lens area—provided better left eye protection than the FSL models. The FSBL models provided shielding rates of approximately 50% or higher at a distance of 1.3 cm with 0.5 or 0.75 mm Pb thickness, whereas the shielding rates of FSL models exceeded 50% only at a Pb thickness of 0.5 or 0.75 mm and a distance of 0.5 cm. These results are in line with those of Silva et al., who reported that protective effects for the left eye were improved by the addition of shields to the lower edge of the front lenses.^64^ This agreement with Silva et al. may be attributed to the behavior of scattered photons during IR procedures, which can reach the IR physician's eyes from below through the gap between the Pb lenses and cheeks.^51^ In this study, the spatial distributions of scattered photons incident on the eyes were visualized at several planes, showing that photons entering the left eye were shielded by the bottom shields. Our findings provide further evidence that bottom shields are effective at protecting the left eye from these photons. Furthermore, the fact that FSBL models with 0.07 mm Pb lenses provided higher shielding rates than FSL model with 0.75 mm Pb lenses indicates that optimization of the lens shape can improve these protective effects.

According to an ICRP report, the conservative dose reduction factor (DRF; ratio of the dose without protection divided by the dose with Pb eyewear) is 2 for 0.75 mm Pb eyewear with side shields or wraparound type eyewear.^2^ This conservative DRF is based on Magee et al.^37^ As a DRF of 2 is equivalent to a shielding rate of 50%, left eye protection provided by the FSBL models (with 0.07, 0.5, or 0.75 mm Pb lens thickness) was better than the reference value at a distance of 1.0 cm or less. A previous clinical study reported a shielding rate of nearly 60% for 0.07 mm Pb eyewear (original model for FSBL model).^65^ In light of these findings, the shielding rate of 49–57% for our FSBL model with 0.07 mm Pb thickness and a distance of 0.5–1.0 cm appears reasonable.

In contrast to our findings of high protection for the left eye, the shielding rates for the right eye were <12% and remained low regardless of presence of bottom shields and distance. Although increases in shielding rates were observed with increasing lead thickness, this parameter was not the dominant factor affecting shielding rates for the right eye. These findings were further supported by the spatial distribution of photons, which demonstrated that scattered photons reached the right eye through the gap around the nose. Previous MC studies have reported low shielding rates for the right eye with Pb eyewear that has side shields, such as the FSL model.^52^ Koukorava et al. reported that wraparound and Pb eyewear with side shields reduced the right eye dose by 24% and 36%, respectively.^51^ Given our findings and those of previous work, the necessity for improved eyewear designs for right‐eye protection should be evaluated through clinical dose assessments.

### 4.2 Effect of variation in forehead gap on eye lens dose

Several studies report that the dose to the eye closer to the X‐ray source (i.e., the left eye) is higher than that to the other eye.^41^, ^52^ Accordingly, in clinical practice, the personal dosimeter is typically placed near the collar of the lead apron or near the left eye.^66^, ^67^, ^68^ For this reason, MC studies of conservative dosimetry methods tend to use the dose to the sensitive volume of the left eye as a reference.^69^ Consistent with these findings, the doses to the right eye were generally lower than those to the left eye for our FSL model. Notably, the left eye dose approached the right eye dose as the distance between the front Pb lens and forehead decreased.

However, for our FSBL models with 0.5 or 0.75 mm Pb lenses, the left eye dose was lower than the right eye dose at a distance of 1.3 cm or less. It is possible that the eye lens dose was underestimated. As we used a single PA irradiation setup in our simulation, the evaluation of the influence of Pb eyewear models on dose management is limited. Further investigations from the perspective of dose monitoring are thus warranted.

### 4.3 Eye lens protective effects afforded by prescription glasses

According to a previous phantom study, eye protective efficiency increases when prescription glasses made of plastic are worn under Pb eyewear.^40^ This study reported that 1.8 mm thick plastic lenses shielded 7% of the dose by 80 kVp incident photons and 5 mm thick plastic frames shielded 14%, indicating modest protective effects. However, our findings indicated little difference in the shielding rates for the left eye with or without the presence of prescription glasses with titanium frames and plastic lenses. Although the prescription glasses appeared to protect the right eye, the shielding rate of the prescription glasses alone was only 3.4%.

One possible explanation of this discrepancy is the thinner, narrower frames of the prescription glasses used in our simulation. Multiple studies have highlighted the importance of covering a large area of the eyes to achieve better protection.^50^, ^70^

In this study, for the right‐eye side, photons were observed to be incident on the prescription glasses through the gap around the nose. However, most photons were incident on the plastic lens material (low atomic number), and thus their contribution to protection was considered small. In contrast, photons incident on the left‐eye side were largely shielded by the front and side shields, and relatively a small number of photons reached the prescription glasses. Therefore, contribution to left‐eye protection was considered minor. As prescription glasses vary widely in their frame dimensions, additional simulations using multiple glasses designs, X‐ray irradiation angles, and X‐ray energies are necessary to clarify the contribution of prescription glasses to eye protection.

### 4.4 Limitations and future directions

Our study is subject to several limitations that could be addressed in future studies. We evaluated the protective effects of fit‐over glasses in relative terms. Therefore, further studies involving eye lens dosimetry are needed.^71^ The combination of X‐ray tube voltage and additional filtration was fixed at 80 kVp with 2.9 mm Al filtration. The primary X‐ray direction was limited to PA, which is commonly used in clinical settings. In this study, the orientation of the operator's head was fixed in the frontal position, assuming a typical situation in which operator faces a front‐mounted monitor. Therefore, the orientation will be varied in future studies. Phantom experiments will also be performed to validate the shielding rates. In this study, the patient was represented by a simplified model based on the dimensions of an abdominal CTDI phantom, which may not fully account for patient‐derived scattered radiation depending on the field of view.

Although eyewear with a Pb‐equivalent thickness <0.5 mm does not comply with International Electron Commission standards and cannot be used in Europe,^72^ our finding that FSBL models with 0.07 mm Pb lenses provided higher shielding rates than any FSL model provides useful information for improving eye protection without increasing weight. However, even the FSBL models exhibited low shielding rates for the right eye. Thus, the need for improved eyewear designs should be further evaluated through clinical dose assessments. It should also be noted that the dose deposited in the sensitive volume of the right eye was higher than that of the left eye in the FSBL models with 0.5 and 0.75 mm Pb lenses. Since in clinical practice, the dose near the left eye is typically measured, validation of monitoring methods is necessary to conservatively estimate the eye lens dose when using FSBL models.

## 5 CONCLUSION

This MC study evaluated the eye protection afforded by Pb eyewear with the presence of prescription glasses and an increasing distance between the Pb lenses and forehead. In this study, the contribution of prescription glasses to eye lens protection was negligible. Our findings showed that FSBL models provided better protection for the eye near the X‐ray source (left eye) than conventional side‐shield models. For FSBL models with 0.5 and 0.75 mm Pb thickness, the shielding rate for the left eye exceeded 76% at 1.0 cm and 50% at 1.3 cm. Notably, the FSBL models with 0.07 mm Pb thickness provided better protective effects than the conventional model with 0.75 mm Pb thickness. Taken together, these findings provide evidence of the effectiveness of bottom shields in improving shielding rates during PA projections and can help IR physicians select Pb eyewear that accommodates their individual prescription glasses. Moreover, our findings suggest that conventional models can be improved in terms of both weight and protective effects of the left eye by optimizing the lens shape. In contrast, the shielding rates for the right eye remained low because of the gap around the nose. Thus, the need for new eyewear designs with a shielded covering over the nose should be further evaluated through clinical dose assessments.

## CONFLICT OF INTEREST STATEMENT

The authors declare no conflicts of interest.

## Data Availability

Data are available from the corresponding author upon reasonable request.
